# *Ehrlichia chaffeensis* DapE Is Essential for Intracellular Growth and Represents a Promising Therapeutic Target

**DOI:** 10.3390/microorganisms14091889

**Published:** 2026-08-25

**Authors:** Mengyao Wang, Yuhong Zhou, Mengxiao Li, Shanhua Qin, Ziyue Qi, Meifang Li, Nan Yang, Yi Zhou, Xiaoya Wei, Yujie Zhang, Zhonghui Yang, Zhihui Cheng

**Affiliations:** 1 The Key Laboratory of Molecular Microbiology and Technology, Ministry of Education, Nankai University, Tianjin 300071, China; 2120231469@mail.nankai.edu.cn (M.W.); 2120231471@mail.nankai.edu.cn (Y.Z.); limengxiao1221@163.com (M.L.); qinshanhua2000@163.com (S.Q.); 2110474@mail.nankai.edu.cn (Z.Q.); 2120211156@mail.nankai.edu.cn (M.L.); 2120211170@mail.nankai.edu.cn (N.Y.); 2210264@nankai.edu.cn (Y.Z.); yyaxiaowei@mail.nankai.edu.cn (X.W.); zyj17867400215@163.com (Y.Z.); 2120251561@mail.nankai.edu.cn (Z.Y.); 2 Department of Microbiology, College of Life Sciences, Nankai University, Tianjin 300071, China

**Keywords:** *Ehrlichia chaffeensis*, DapE, antibiotics, IL-8, innate immune responses

## Abstract

*Ehrlichia chaffeensis* is an obligate intracellular bacterium that proliferates within monocytes or macrophages and causes human monocytic ehrlichiosis (HME), an emerging life-threatening zoonosis. Doxycycline is the choice of treatment for HME, yet it has prominent side effects. Host cells lack the lysine biosynthetic pathway; thus, the enzymes in this pathway are essential for bacterial growth and recognized as potential targets for the development of novel antibiotics. Here, we demonstrated that inhibitors targeting DapE, which is a key enzyme in the lysine biosynthetic pathway, especially disulfiram, effectively inhibit *E. chaffeensis* infection and intracellular growth. Through complementation experiments and peptide nucleic acid-mediated *dapE* knockdown, we showed that DapE in *E. chaffeensis* is functional and essential for bacterial intracellular growth. Using purified recombinant protein, we found that DapE induces IL-8 expression in host cells. Finally, we identified that NtrX, the response regulator of the two-component system NtrY/NtrX, regulates *dapE* expression using an electrophoretic mobility shift assay and a reporter assay. Our findings deepen the understanding of *E. chaffeensis* pathogenesis as well as illustrate that DapE in *E. chaffeensis* is a potential therapeutic target for the development of novel HME treatments.

## 1. Introduction

*Ehrlichia chaffeensis* is an obligate intracellular bacterium belonging to the order Rickettsiales, the family *Anaplasmataceae* [1]. Transmitted by ticks, its main hosts are white-tailed deer [2]. *E. chaffeensis* infects human monocytes or macrophages, causing human monocytic ehrlichiosis (HME) [3]. The early-stage symptoms of HME include acute fever, headache, myalgia, anorexia, and chills, which readily lead to misdiagnosis and delayed treatment [4]. In addition, the disease poses a greater threat to immunocompromised individuals and the elderly [3]. HME is associated with a notably high hospitalization rate of 83.2% in symptomatic cases, and an overall fatality rate of 11.6%, which is significantly elevated in immunocompromised patients (16.3%) compared with immunocompetent individuals (9.9%) [5]. HME can be treated with doxycycline, a broad-spectrum antibiotic [3]; however, this drug has substantial side effects and is contraindicated in specific populations such as children, pregnant women, the elderly, and immunocompromised patients [6]. Given the considerable overlap between doxycycline-ineligible populations and those at high risk of HME severe symptoms, the development of novel therapeutic drugs is urgently required.

*E. chaffeensis* infection induces the expression of IL-1β, IL-8, and IL-10 at both mRNA and protein levels in human monocytic THP-1 cells [7]. *E. chaffeensis* does not possess canonical pathogen-associated molecular patterns (PAMPs), including LPS, peptidoglycan, flagella, pili, and capsules [8]. Cytokine induction by *E. chaffeensis* is dependent on other types of molecules [9]. *E. chaffeensis* has an incomplete peptidoglycan biosynthetic pathway [8]. Ech_1067, a key enzyme of this pathway and a penicillin-binding protein, is expressed on the bacterial membrane and acts as a signal recognized by host innate immunity [9]. Ech_1067 can induce the expression of multiple cytokines, including IL- 1β, IL-8 and IL-10, in host cells [9]. The other proteins in the incomplete peptidoglycan biosynthetic pathway might also play roles in the induction of the expression of cytokines.

The lysine biosynthetic pathway is widely present in most bacteria [10], and *E. chaffeensis* harbors an intact lysine biosynthetic pathway [11]. Host cells inherently lack this pathway [12], making its preservation critical for the intracellular growth of this obligate intracellular bacterium. This pathway provides diaminopimelic acid (DAP) for bacterial peptidoglycan biosynthesis and lysine production. DapE, a succinyl-diaminopimelic acid desuccinylase, specifically catalyzes the desuccinylation of N-succinyl-L, L-diaminopimelic acid (L,L-SDAP) to generate L,L-diaminopimelic acid (L,L-DAP) [13]. In the subsequent step of the lysine biosynthetic pathway, L,L-DAP is isomerized to meso-diaminopimelic acid (meso-DAP) by the downstream enzyme DapF [14]. The meso-DAP is either converted into L-lysine or employed to synthesize peptidoglycan. Furthermore, the amino acid sequence of DapE is highly conserved across bacterial species [15], rendering it a promising target for the development of novel antibiotics [10].

A variety of compounds have been identified as specific inhibitors of DapE, including penicillamine [10], captopril [16] and disulfiram [17], whose inhibitory activities and mechanisms have been experimentally verified, providing a basis for the development of DapE-targeted antibacterial agents. Penicillamine acts as a competitive inhibitor of *Haemophilus influenzae* DapE (HiDapE) with strict stereoselectivity. Its thiol group interacts with the binuclear Zn(II) center in the active site [10]. Captopril, an approved angiotensin-converting enzyme inhibitor, exhibits competitive inhibitory activity against both HiDapE and *Acinetobacter baumannii* DapE (AbDapE). Structural studies confirmed that captopril binds to the enzyme active site via its thiol group and shows dose-dependent antimicrobial activity against *Escherichia coli* [16]. Disulfiram, an approved anti-alcoholism drug, effectively inhibits DapE from *Enterococcus faecium* and *E. coli* by reducing enzyme thermal stability [17]. Its favorable clinical safety and low repurposing cost make it a promising candidate for the development of antibiotics [17].

In this study, we aimed to investigate the effects of penicillamine, captopril and disulfiram on *E. chaffeensis* infection and intracellular growth, along with DapE’s biological functions, the interaction with host immune responses, and the expression regulatory mechanisms.

## 2. Materials and Methods

### 2.1. Bacteria and Cell Culture, Plasmids, and Primers

The Arkansas strain of *E. chaffeensis* was used as the experimental strain in this study. It was propagated in human acute leukemia THP-1 cells cultured in RPMI 1640 medium (Cytiva, Marlborough, MA, USA) supplemented with 2 mM L-glutamine and 10% fetal bovine serum (FBS, Every Green, Huzhou, Zhejiang, China). The incubation was performed at 37 °C with 5% CO_2_, as previously described [18]. *E. coli* strains DH5α and BL21 (DE3) for DNA cloning and protein expression were cultured in Luria–Bertani (LB) broth with corresponding antibiotics when needed. *E. coli* strain BW25113, which harbors the plasmid pKD46 and is used for gene knockout and gene complementation, was cultured in LB broth with the addition of either kanamycin (50 µg/mL) (Yuanye, Shanghai, China) or diaminopimelic acid (DAP, 0.1 mg/mL) (Yuanye, Shanghai, China) based on experimental requirements. All cell lines and bacterial strains are listed in Supplementary Table S1. Plasmids and primers used for λ-Red recombination, gene complementation, gene cloning and quantitative reverse transcription PCR (qRT-PCR) are summarized in Supplementary Table S2. The THP-1 cell line was obtained from Fuheng Biotechnology (Shanghai, China, FH0112).

### 2.2. Isolation of Host Cell-Free E. chaffeensis

A total of 2 × 10^7^ THP-1 cells infected with *E. chaffeensis* (infection rate > 90% and large inclusions) were harvested by centrifugation at 600× *g* for 5 min at room temperature. The cell pellet was resuspended in 6 mL ice-cold 1 × SPK buffer (pH 7.4) containing 200 mM sucrose, 50 mM potassium phosphate and 2 mM L-glutamine [19]. The host cell membrane was disrupted by passing the cell suspension through a 23-gauge needle 30 times using a syringe on ice. Undisrupted cells and cell debris were removed by centrifugation at 1000× *g* for 5 min at room temperature. The resulting supernatant was centrifuged at 10,000× *g* for 10 min at 4 °C to harvest bacteria, which was then used for drug treatment and peptide nucleic acid transfection.

### 2.3. Quantitative RT-PCR

Quantitative RT-PCR experiments were performed according to the previously published protocol with minor modifications [20]. Total RNA was extracted from all samples using a TransZol Up Plus RNA Kit (TransGen Biotech, Beijing, China). The isolated RNA was then reverse-transcribed into complementary DNA (cDNA) with the HiScript III RT SuperMix for qPCR (+gDNA wiper) (Vazyme Biotech, Nanjing, Jiangsu, China). Quantitative real-time PCR was carried out on a StepOnePlus Real-Time PCR System (Applied Biosystems, Woburn, MA, USA) using gene-specific primers (Supplementary Table S2) and the PerfectStart Green qPCR SuperMix (TransGen Biotech). The assay was designed to target transcripts of *E. chaffeensis* 16S rRNA, *dapE*, human *GAPDH*, IL-1β, IL-8, and IL-10. Relative expression levels of IL-1β, IL-8, and IL-10 were normalized against the housekeeping gene *GAPDH*. The transcript level of *dapE* in *E. chaffeensis* was calibrated using bacterial 16S rRNA as the internal reference. Bacterial loads were calculated based on bacterial 16S rRNA levels normalized against human *GAPDH* levels.

### 2.4. Drug Treatment of E. chaffeensis

The drug treatment of *E. chaffeensis* was carried out according to the previously published protocol with minor modifications [21]. *E. chaffeensis*-infected THP-1 cells were seeded in 6-well plates at a total volume of 2 mL per well, at a density of approximately 5 × 10^5^ cells/mL. The cells were incubated with different concentrations of disulfiram (3.5 μM, 7 μM), captopril (40 μM, 100 μM), or penicillamine (3 μM, 6 μM) (all from MCE, Shanghai, China), respectively, at 37 °C for 48 h.

The drug pretreatment of *E. chaffeensis* was carried out according to the previously published protocol with minor modifications [21]. Purified *E*. *chaffeensis* was resuspended in 1 × SPK buffer and pretreated with different concentrations of captopril (40 μM, 100 μM) or disulfiram (3.5 μM, 7 μM), respectively, at 37 °C for 30 min. Then bacteria were washed with the culture medium and added to 6-well plates pre-seeded with THP-1 cells (at a density of approximately 5 × 10^5^ cells/mL, with a total volume of 2 mL per well). After incubation for 48 h at 37 °C, all cells in each well were harvested. Bacterial numbers were determined using qRT-PCR.

### 2.5. λ-Red Recombination for DapE Knockout in E. coli BW25113

*E. coli* BW25113/pKD46 was cultured overnight at 30 °C in LB broth, then subcultured in fresh LB broth. The culture was shaken at 30 °C to OD_600_ 0.2–0.3, then supplemented with 0.4% (*v*/*v*) filter-sterilized L-arabinose and induced for 2 h, following the standard λ-Red recombineering protocol [22,23]. Induced cultures were chilled on ice for 20 min, centrifuged at 4000× *g* for 10 min at 4 °C, and the pellet was washed three times with 1 mL ice-cold sterile 10% (*v*/*v*) glycerol. The final pellet was resuspended in 100 μL 10% glycerol to prepare electrocompetent cells. Using the pKD13 plasmid as the template, PCR was performed with primers specifically designed to contain the recombinant sequences of the *E. coli* BW25113 *dapE* gene at their 5′ ends, resulting in a DNA fragment flanked by FRT sequences. 150 ng of amplicon was incubated with the competent cells on ice for 30 min, then electroporated in a pre-chilled 2 mm cuvette. Immediately after electroporation, 1 mL LB broth supplemented with 0.4% L-arabinose and 0.1 mg/mL DAP was added, then incubated overnight at 30 °C. The culture was centrifuged at 5000× *g* for 5 min at room temperature. The pellet was resuspended in 100 μL LB broth and spread onto LB agar plates supplemented with 50 μg/mL kanamycin and 0.1 mg/mL DAP, then incubated at 30 °C for 48 h. Putative colonies were screened by colony PCR with *dapE*-specific primers (Supplementary Table S2), then cultured on LB plates with or without 0.1 mg/mL DAP for knockout verification. Verified colonies were cultured overnight at 42 °C to remove pKD46.

### 2.6. Expression and Purification of Recombinant Proteins

Recombinant proteins were expressed and purified according to the previously published protocol with slight adjustments [19]. Specific primers (Supplementary Table S2) were used to amplify the *dapE*-encoding region. The resulting PCR product was inserted into the pET-33b(+) expression vector to produce N-terminally His-tagged recombinant DapE (rDapE). The ligation products were introduced into *E. coli* DH5α competent cells, and the recovered plasmids were verified by sequencing. Sequence-confirmed constructs were transformed into *E. coli* BL21 (DE3) cells. Recombinant protein expression was induced using 0.1 mM isopropyl-thio-β-D-galactoside (IPTG, Solarbio, Beijing, China) at 37 °C for 4 h. The recombinant protein was isolated via Ni^2+^-affinity chromatography and subsequently dialyzed against the storage buffer containing 10 mM Tris-HCl (pH 7.5) and 1 mM DTT (Solarbio). Similarly, the recombinant NtrX DNA-binding domain (rNtrX DBD), also bearing an N-terminal His-tag, was expressed using 1 mM IPTG induction at 20 °C for 5 h and purified using the identical workflow.

### 2.7. Western Blotting

Western blotting analysis was carried out following the previously published protocol with slight modifications [24]. For detection of recombinant rNtrX and rNtrYHKD proteins, samples were mixed with 5 × SDS loading buffer and heated at 100 °C for 10 min. Proteins were then resolved via 12% SDS-PAGE and electrotransferred to PVDF membranes (Millipore, Cork, Ireland). The membranes were probed with the mouse anti-His monoclonal antibody (1:1000, Merck, Darmstadt, Germany, 05-949) at room temperature for 1 h, followed by three washes with PBST (137 mM NaCl, 2.7 mM KCl, 10 mM Na_2_HPO_4_·7H_2_O, 2 mM KH_2_PO_4_, 0.1% (*v*/*v*) Tween 20). Next, the HRP-linked goat anti-mouse IgG H&L secondary antibody (1:1000, Promega, Madison, WI, USA, W4021) was applied at room temperature for 1 h, followed by three PBST washes. Target protein bands were visualized using an Immobilon Western Kit (Millipore) and a ChemiDoc XRS+ Imaging System (BIO-RAD, Hercules, CA, USA).

### 2.8. Treatment of THP-1 Cells with DapE

Treatment of THP-1 cells with rDapE was carried out following the previously published protocol with slight modifications [9]. THP-1 cells were seeded in 6-well plates at a density of 5 × 10^5^ cells/mL, pretreated with 20 μg/mL polymyxin B (PB, MCE) at 37 °C for 24 h, and then incubated with rDapE or rNtrX DBD (as a negative control) at different concentrations (0.5, 2, 4 μg/mL) for another 2 h at 37 °C. The expression levels of cytokines were determined using qRT-PCR as described above.

### 2.9. Peptide Nucleic Acid Transfection

PNA-mediated gene knockdown targeting the *dapE* gene in *E. chaffeensis* was conducted following the previously published protocol [18]. Two PNA oligomers, including an antisense PNA that specifically binds to the 1071–1086 bp sequence downstream of the *dapE* start codon (designated as DapE PNA) and a non-specific scrambled control PNA (CTL PNA), were commercially synthesized by TAHEPNA Biotechnology Co., Ltd. (Hangzhou, Zhejiang, China). Detailed sequence information for both PNAs is listed in Supplementary Table S2. Custom *dapE* single-stranded RNA fragment (ssRNA) was synthesized by GENTLEGEN (Suzhou, Jiangsu, China) for PNA binding verification (Supplementary Table S2). Briefly, 40 pmol of *dapE* ssRNA was mixed with 20 pmol of DapE PNA or CTL PNA in a 15 μL reaction system containing 250 mM Tris-HCl (pH 7.2). Hybridization was conducted on a BIO-GENER GE4852T thermal cycler (Bio-Gener, Hangzhou, Zhejiang, China): 95 °C for 3 min, followed by 40 cycles from 95 °C to 55 °C, each for 20 s. Samples were separated via 12% native PAGE, and nucleic acid bands were visualized with GelGreen staining (Biomed, Beijing, China) using a ChemiDoc XRS+ Imaging System.

For *dapE* knockdown, 3 μg of DapE PNA or CTL PNA (dissolved in nuclease-free water) was mixed with 100 μL of purified *E. chaffeensis* suspension in 0.3 M sucrose solution. After 15 min of ice incubation for sufficient binding, electroporation was performed in a 2 mm cuvette using a BIO-RAD Gene Pulser Xcell system (BIO-RAD) with parameters of 2000 V, 25 μF, 400 Ω and 10 ms pulse duration. Electroporated *E. chaffeensis* was transferred to T25 flasks and used to infect 5 × 10^5^ THP-1 cells. After 2 h of incubation at 37 °C with shaking every 15 min to facilitate infection, cells were harvested at 48 h post-infection (p.i.). The transcriptional level of *dapE* was detected by qRT-PCR to evaluate the knockdown efficiency of DapE PNA.

### 2.10. Construction of the lacZ Transcriptional Fusion and β-Galactosidase Activity Assay

Construction of the *lacZ* transcriptional fusion and β-galactosidase activity assay were performed as described previously with minor modifications [25]. A 302 bp DNA fragment corresponding to the *dapE* promoter region was amplified and cloned upstream of the *lacZ* gene in the pACYC184-*lacZ* vector [25]. BL21(DE3) strain expressing rNtrX or rNtrYHKD (negative control) from pET-33b(+) was transformed with the *dapE* promoter-*lacZ* fusion. Protein induction was performed using 1 mM IPTG at 37 °C for 4 h, and β-galactosidase activity was then determined.

### 2.11. Electrophoretic Mobility Shift Assay

The electrophoretic mobility shift assay (EMSA) was carried out according to the previously published protocol with slight adjustments [26]. A 302 bp DNA fragment corresponding to the *dapE* promoter region was amplified. The DNA fragment (30 ng) was incubated with rNtrX DBD in a 20 μL reaction mixture containing 1 mM DTT and 10 mM Tris-HCl (pH 7.5) on ice for 30 min. Subsequently, the samples were subjected to 8% native PAGE, which was pre-run in 1 × TBE buffer for 1 h. After electrophoresis on ice, the gel was stained for 5 min in DNA staining buffer containing the StarStain Red Enhanced Nucleic Acid Dye (GenStar, Beijing, China) diluted at 1:10,000, and visualized using the ChemiDoc XRS+ Molecular Imager.

### 2.12. Cytotoxicity Assay of THP-1 Cells by Calcein AM/PI Double Staining

THP-1 cells were seeded in black 96-well culture plates at a density of 4 × 10^4^ cells/mL, followed by treatment with captopril (40 μM, 100 μM) or disulfiram (4 μM, 8 μM), respectively. The plates were incubated at 37 °C for 48 h. After incubation, the cells were centrifuged at 1000× *g* for 5 min at room temperature, then resuspended in pre-chilled PBS. Cell viability and cytotoxicity were determined using the Calcein/PI Cell Viability/Cytotoxicity Assay Kit (Beyotime Biotechnology, Shanghai, China) according to the previously published protocol [27]. Calcein AM (1000×) and PI (1000×) were diluted at 1:1000 in the assay buffer and mixed thoroughly to prepare the double-staining working solution, which was used with light protection throughout the process. Each well received 100 μL of the Calcein AM/PI double-staining working solution. The plates were then incubated at 37 °C for 30 min. After incubation, the fluorescence signals of each well were directly detected using a Varioskan Flash Multimode Microplate Reader (Thermo Fisher Scientific, Waltham, MA, USA): the fluorescence of Calcein AM (Ex = 494 nm, Em = 517 nm) for viable cells, and the fluorescence of PI (Ex = 535 nm, Em = 617 nm) for dead cells. Cell-free blank wells with only the double-staining working solution were set as the background fluorescence control. The fluorescence values of all test wells were subtracted by the background fluorescence values of blank wells, and the relative fluorescence intensity (RFU) of viable and dead cells in each group was calculated.

### 2.13. Statistical Analysis

All assays were repeated no fewer than three times. GraphPad Prism 10.0 was used for statistical analysis. Two groups were compared via two-tailed Student’s *t*-test, while one-way ANOVA was used for comparisons among three or more groups. Differences with *p* < 0.05 were considered statistically significant.

## 3. Results

### 3.1. E. chaffeensis DapE Is a Potential Antibiotic Target and Effectively Inhibited by DapE Inhibitors

*E. chaffeensis* is an obligate intracellular bacterium that acquires various nutrients from host cells [3]; however, human cells lack the lysine biosynthetic pathway endogenously [28]. *E. chaffeensis* harbors a complete lysine biosynthetic pathway [11]. Since DapE is a key enzyme in this pathway [10], we investigated the effects of three DapE-targeting drugs, disulfiram, captopril and penicillamine, on *E. chaffeensis* infection and intracellular growth.

**Figure 1:**
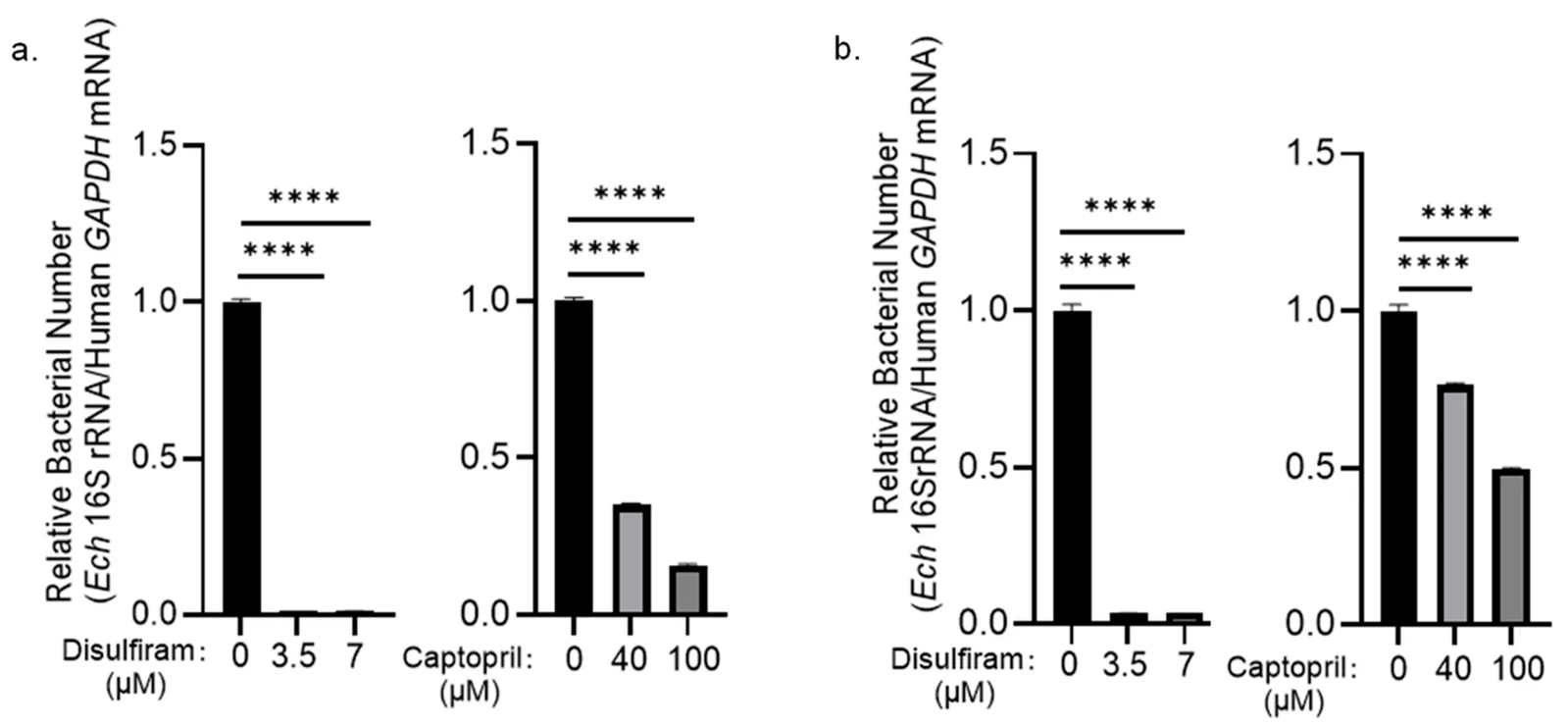
The effects of disulfiram and captopril on *E. chaffeensis* intracellular growth and infection. (**a**) *E. chaffeensis*-infected THP-1 cells were treated with disulfiram (3.5 μM, 7 μM) or captopril (40 μM, 100 μM), respectively, at 37 °C. (**b**) Host cell-free *E. chaffeensis* was pretreated with disulfiram (3.5 μM, 7 μM) or captopril (40 μM, 100 μM), respectively, at 37 °C for 30 min, then used to infect THP-1 cells. At 48 h p.i., total RNA was isolated from individual samples. The load of *E. chaffeensis* was quantified by qRT-PCR and normalized against human *GAPDH* mRNA. Relative levels of bacterial 16S rRNA were normalized to the control group. Results are presented as mean ± SD (*n* = 3). Statistical differences were evaluated by one-way ANOVA (**** *p*  <  0.0001).

Infected cells were treated with different concentrations of each drug, and the bacterial numbers were determined at 48 h p.i. Treatment with disulfiram at 3.5 μM or 7 μM inhibited bacterial intracellular growth by 98.66% and 98.73% (Figure 1a), respectively. Treatment with captopril at 40 μM or 100 μM inhibited bacterial intracellular growth by 65.13% and 84.23% (Figure 1a), respectively. In contrast, penicillamine treatment had no effect on *E. chaffeensis* intracellular growth (Figure S1).

Since penicillamine showed no inhibitory effect, we further evaluated the effects of disulfiram and captopril on *E. chaffeensis* infection. Pretreatment of host cell-free *E. chaffeensis* with 3.5 μM or 7 μM disulfiram reduced *E. chaffeensis* infection by 96.78% and 96.54% (Figure 1b), respectively. Pretreatment with 40 μM or 100 μM captopril reduced bacterial infection by 23.69% and 50.52% (Figure 1b), respectively. Disulfiram and captopril exhibited no cytotoxicity at the concentrations used in this study (Figure S2). These results indicate that *E. chaffeensis* DapE is a potential antibiotic target, and disulfiram exhibits significant inhibitory activity against *E. chaffeensis* infection and intracellular growth.

### 3.2. E. chaffeensis DapE Functions as a Succinyl-Diaminopimelic Acid Desuccinylase

**Figure 2:**
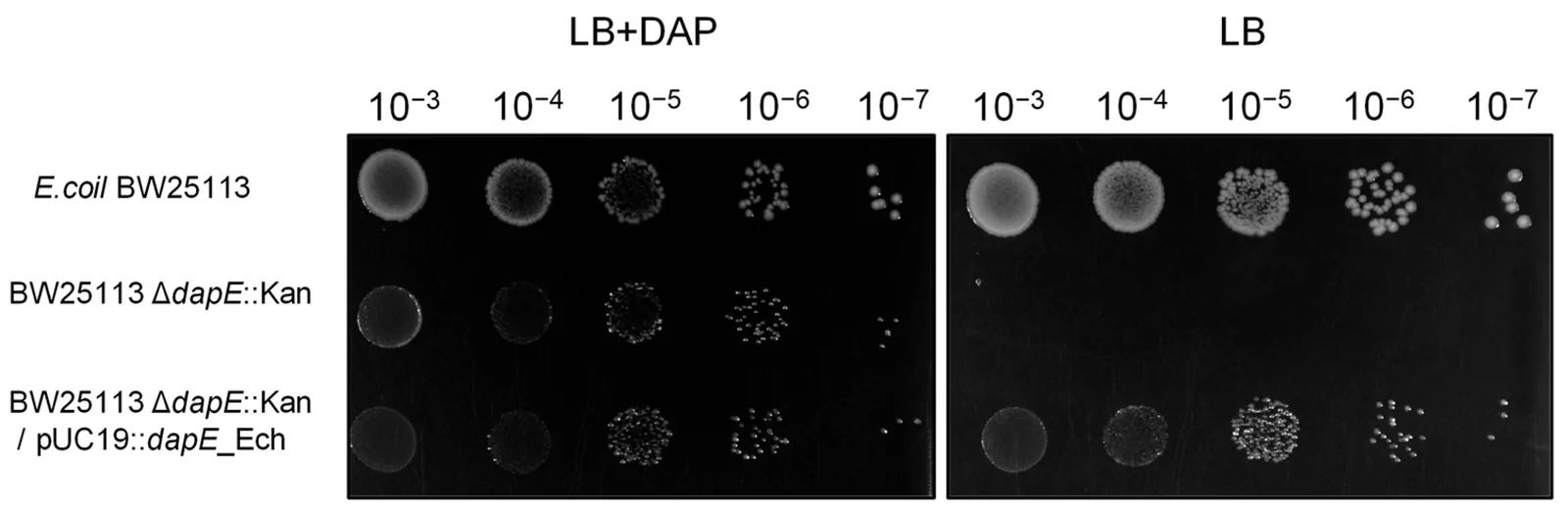
*E. chaffeensis* DapE is a functional succinyl-diaminopimelic acid desuccinylase. *E. coli* BW25113 (wild-type) strain, BW25113 Δ*dapE*::Kan strain, or BW25113 Δ*dapE*::Kan/pUC19::*dapE*_Ech (*dapE*-complemented) strain was serially diluted and plated on LB agar supplemented with DAP (**left panel**) or LB agar only (**right panel**).

Next, we verified whether *E. chaffeensis* DapE is functional. To date, no *dapE* deletion mutant of *E. chaffeensis* has been successfully generated, as conventional bacteriology techniques are not readily applicable for this obligate intracellular bacterium [3]. Thus, we used *E. coli* as a surrogate system to study the function of *E. chaffeensis* DapE. The amino acid sequence of *E. chaffeensis* DapE shows 38% identity and 55% positivity to that of *E. coli* DapE. We first deleted the *dapE* gene in *E. coli* BW25113 via λ-Red recombination. Then we constructed the recombinant plasmid pUC19::*dapE*_Ech harboring the *E. chaffeensis dapE* gene, and electroporated this plasmid into the *E. coli* BW25113 Δ*dapE*::Kan strain for gene complementation. The wild-type *E. coli* BW25113 strain, Δ*dapE* strain, and *dapE*-complemented strain were separately cultured on LB agar plates with or without DAP. The results showed that the wild-type strain and the complemented strain grew normally on LB agar plates without DAP, while the Δ*dapE* strain barely grew, indicating that *E. chaffeensis* DapE is a succinyl-diaminopimelic acid desuccinylase and is crucial for bacterial growth (Figure 2).

### 3.3. E. chaffeensis DapE Induces the IL-8 Expression in THP-1 Cells

**Figure 3:**
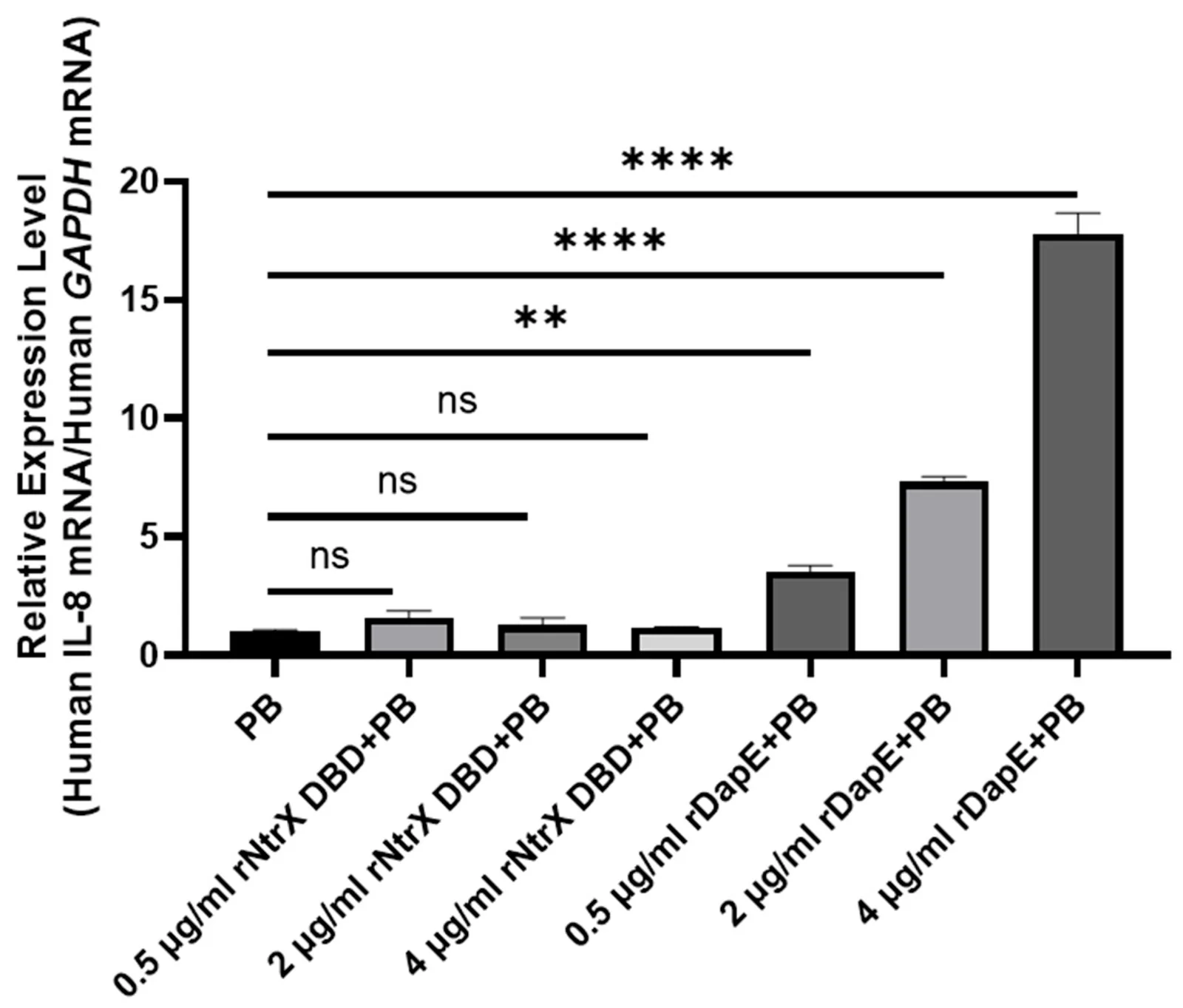
DapE induces IL-8 expression in THP-1 cells. THP-1 cells were seeded in 6-well plates at a density of 5 × 10^5^ cells/mL, followed by incubation with 20 μg/mL PB at 37 °C for 24 h. Subsequently, the cells were treated with rDapE or rNtrX DBD (as a negative control) at different concentrations (0.5, 2, 4 μg/mL) at 37 °C for 2 h. Total RNA was extracted. IL-8 mRNA levels were detected by qRT-PCR and normalized against those of human *GAPDH* mRNA. Relative values were based on the PB treatment group. Results are presented as mean ± SD (*n* = 3). Statistical differences were evaluated by one-way ANOVA (ns = not significant, ** *p* < 0.01, **** *p*  <  0.0001).

Since *E. chaffeensis* infection induces the expression of IL-1β, IL-8 and IL-10 in THP-1 cells [7], we investigated whether *E. chaffeensis* DapE plays a role in cytokine induction. We pretreated THP-1 cells with 20 μg/mL PB at 37 °C for 24 h to block the biological effects of LPS, which might be present in the purified recombinant proteins. The cells were treated with purified rDapE or rNtrX DBD (recombinant NtrX DNA-binding domain) as a negative control at different concentrations (0.5, 2, 4 μg/mL) at 37 °C for 2 h. The purified rDapE or rNtrX DBD showed a single band on the SDS-PAGE gel (Figure S3a,b). We found that treatment with rDapE upregulated IL-8 expression in a dose-dependent manner (Figure 3), while it had no effect on the expression of IL-1β (Figure S4a) or IL-10 (Figure S4b), indicating that *E. chaffeensis* DapE induces IL-8 expression in host cells. The induction specificity was confirmed by the results that no change in IL-8 expression was detected when THP-1 cells were treated with rNtrX DBD using the same method (Figure 3).

### 3.4. DapE Is Essential for E. chaffeensis Intracellular Growth

**Figure 4:**
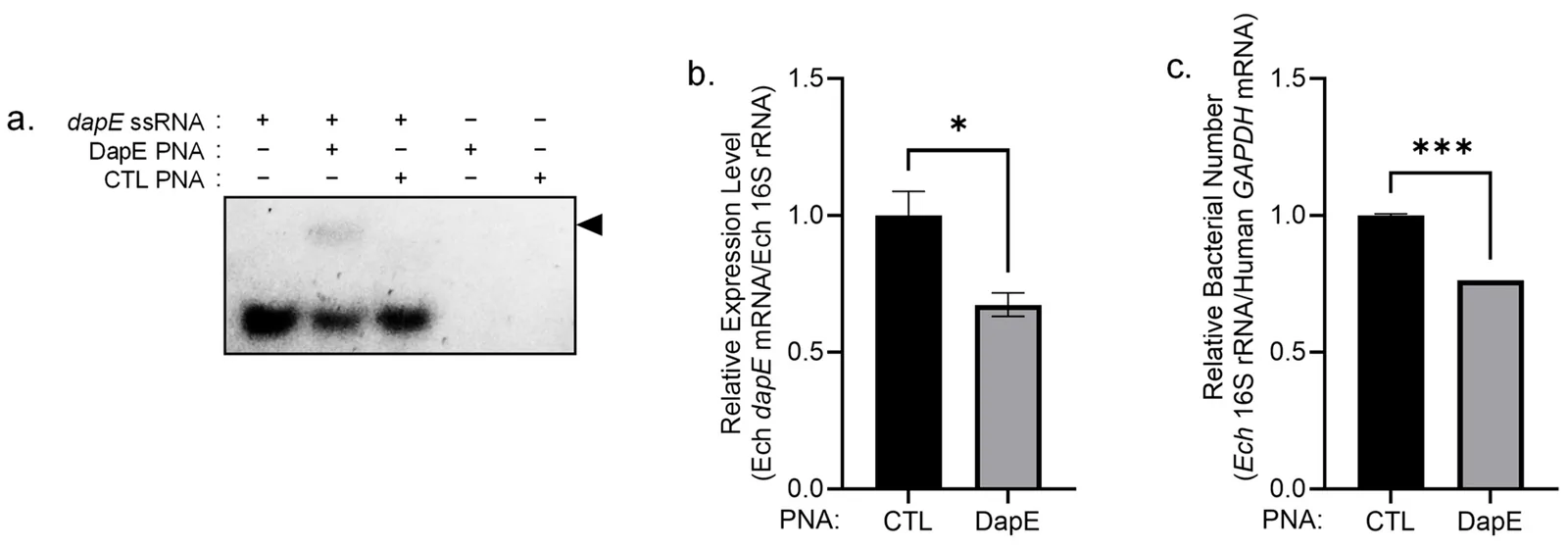
Knockdown of *dapE* in *E. chaffeensis* by PNA inhibits bacterial intracellular growth. (**a**) 40 pmol *dapE* ssRNA probe was annealed alone or in the presence of 20 pmol DapE PNA or CTL PNA, as the temperature gradually decreased from 95 °C to 55 °C. Equal amounts of each PNA alone were included as controls under identical conditions. An arrowhead marks the shifted band corresponding to the PNA-RNA complex. (**b**) qRT-PCR was used to detect the *dapE* expression at 48 h p.i. using *E. chaffeensis* 16S rRNA as the internal reference. Relative levels were based on the CTL PNA-transfected bacteria. Results are presented as mean ± SD (*n* = 3). Statistical differences were evaluated by Student’s *t*-test (* *p* < 0.05). (**c**) At 48 h p.i., bacterial abundance was examined using qRT-PCR. *E. chaffeensis* 16S rRNA was normalized against human *GAPDH* mRNA, and relative values were based on the CTL PNA group. Results are presented as mean ± SD (*n* = 3). Statistical differences were evaluated by Student’s *t*-test (*** *p* < 0.001).

To investigate the role of DapE in *E. chaffeensis* intracellular growth, we performed peptide nucleic acid (PNA)-mediated knockdown targeting the *dapE* gene. PNA is a DNA mimic that can bind single-stranded and double-stranded DNA as well as RNA with high affinity and specificity, thereby suppressing transcription and translation [29]. We designed DapE PNA that specifically binds to the *dapE* gene in the 1071–1086 bp region, calculated from the translational start codon. Its specificity was verified, as DapE PNA bound to *dapE* single-stranded RNA (*dapE* ssRNA, a synthetic mRNA fragment spanning the position of 1063–1093 bp from the start codon) (Figure 4a). When host cell-free *E. chaffeensis* was transfected with DapE PNA, the *dapE* mRNA level dropped markedly at 48 h p.i. (Figure 4b). Moreover, the intracellular growth of bacteria treated with DapE PNA was notably inhibited compared with that of the CTL PNA group (Figure 4c), indicating that DapE is essential for *E. chaffeensis* intracellular growth.

### 3.5. The Expression of dapE Is Regulated by NtrX in E. chaffeensis

**Figure 5:**
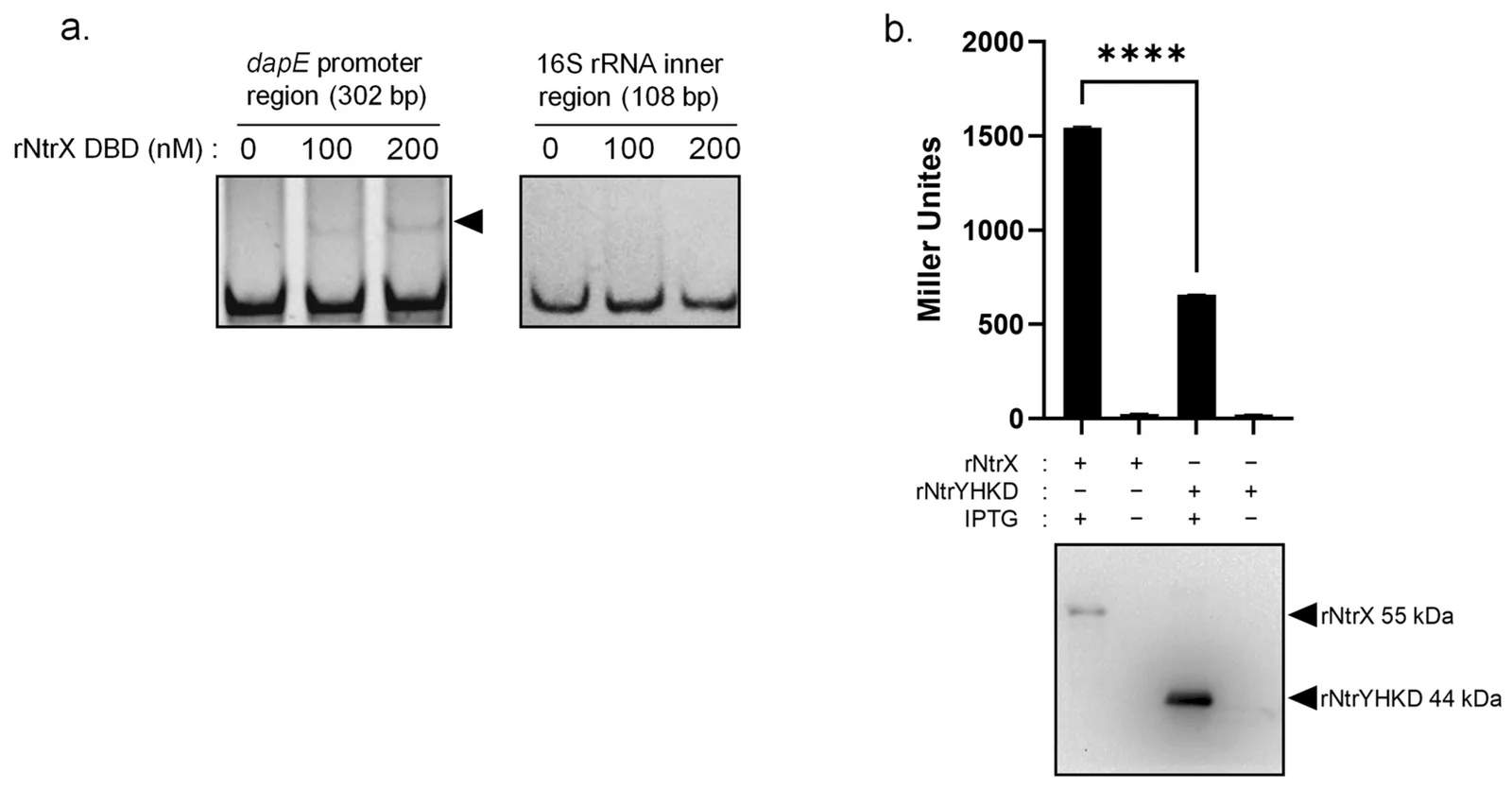
NtrX regulates the *dapE* expression in *E. chaffeensis*. (**a**) NtrX binds to the *dapE* promoter. 50 ng DNA probe was incubated alone, with 100 nM or 200 nM rNtrX DBD on ice for 30 min. The shifted band is marked with an arrowhead. The 16S rDNA fragment serves as a negative control. The probe length (bp) is labeled above each panel. (**b**) NtrX activates the *dapE* expression. *E. coli* strain containing pET-33b(+) encoding rNtrX or rNtrYHKD was transformed with the *dapE* promoter-*lacZ* fusion. Following IPTG-induced expression of rNtrX and rNtrYHKD, β-galactosidase activity was detected. The upper panel presents activity values in Miller Units. Results are presented as mean ± SD (*n* = 3). Statistical differences were evaluated by one-way ANOVA (**** *p*  <  0.0001). The lower panel is Western blotting of samples from the β-galactosidase assay showing the expression of rNtrX and rNtrYHKD.

NtrX is involved in the regulation of amino acid metabolism in *E. chaffeensis*, including the arginine and glutamine biosynthetic pathways [21,25]. ArgD, whose expression is regulated by NtrX [21], is a key enzyme in the arginine biosynthetic pathway and is also involved in the lysine biosynthetic pathway. In the arginine biosynthetic pathway, ArgD uses N-acetyl-L-ornithine and 2-oxoglutarate as substrates to generate L-glutamate-5-semialdehyde and L-glutamate via a transamination reaction [30]. In the lysine biosynthetic pathway, ArgD takes N-succinyl-2-amino-6-ketopimelate as the substrate and produces N-succinyl-L,L-diaminopimelic acid (L,L-SDAP), which is exactly the specific substrate of DapE, through the same transamination reaction [30]. We hypothesized that NtrX might also regulate the expression of *dapE*. To explore whether NtrX interacts directly with the *dapE* promoter, we performed EMSA using the recombinant NtrX DNA-binding domain (rNtrX DBD). The purified rNtrX DBD protein showed a single band on SDS-PAGE (Figure S3b). Incubation of the probe derived from the *dapE* promoter with rNtrX DBD resulted in a clear band shift (Figure 5a). Binding specificity was confirmed by the absence of a shifted band when a 16S rDNA internal fragment was used as the probe (Figure 5a). These findings demonstrate that NtrX directly binds to the *dapE* promoter in *E. chaffeensis*.

To examine whether rNtrX activates *dapE* expression, we inserted the 302 bp *dapE* promoter from *E. chaffeensis* upstream of promoter-less *lacZ* in the pACYC184-*lacZ* vector. *E. chaffeensis dapE* promoter-*lacZ* fusion was then transformed into *E. coli* BL21(DE3) expressing *E. chaffeensis* rNtrX or *E. chaffeensis* NtrY histidine kinase domain (rNtrYHKD) as a negative control. After induction with 1 mM IPTG at 37 °C for 4 h, β-galactosidase activity was determined. The expression of rNtrX induced by IPTG resulted in a significant increase in β-galactosidase activity compared with that of rNtrYHKD (Figure 5b), indicating that NtrX activates the *dapE* expression.

## 4. Discussion

In this study, we found that the *E. chaffeensis* DapE enables the *E. coli* △*dapE* mutant to grow on medium without DAP. Knockdown of *dapE* in *E. chaffeensis* with PNA transfection significantly inhibited bacterial infection and intracellular growth in THP-1 cells. These results demonstrate that *E. chaffeensis* DapE is functional and essential. As a key adaptive strategy for immune escape, *E. chaffeensis* evades detection by host cell pattern recognition receptors (PRRs), such as Toll-like receptors (TLRs) and nucleotide-binding oligomerization domain (NOD)-like receptors (NLRs), which trigger robust innate immune responses, by lacking a complete peptidoglycan biosynthetic pathway and thus containing no peptidoglycan in its cell wall [31,32]. Although this bacterium does not synthesize peptidoglycan [8], it retains an intact lysine biosynthetic pathway [11], in which DapE acts as a pivotal enzyme. The lysine biosynthetic pathway serves as a major source of lysine, an essential amino acid required for bacterial protein synthesis and all core intracellular physiological processes, for *E. chaffeensis* [8]. Since human cells lack the lysine biosynthetic pathway [10], DapE emerges as a highly promising core therapeutic target for the treatment of HME. Currently, research on DapE inhibitors is mainly confined to the enzymatic level, with the primary focus on evaluating their in vitro inhibitory activity against the DapE protein, rather than investigating their functional effects at the bacterial cellular level [28]. To date, the well-documented bactericidal study is that of captopril, which has been shown to effectively inhibit the growth of *E. coli* and form distinct inhibition zones [16].

For intracellular bacteria such as those belonging to the order Rickettsiales, which are classified into three groups based on the presence or absence of peptidoglycan, the lysine biosynthetic pathway is highly conserved across all species, despite the fact that some bacteria lack a functional peptidoglycan biosynthetic pathway [33]. Therefore, targeted inhibition of the lysine biosynthetic pathway holds promise as a potential universal therapeutic strategy for combating Rickettsial pathogens and possibly even treating infections caused by a broad range of intracellular bacteria. In addition to DapE, other enzymes in the lysine biosynthetic pathway, including Asd, DapA, DapB, DapD, ArgD, DapF, and LysA, also exhibit considerable research potential. Currently, inhibitor studies targeting these enzymes are mostly based on structure-based drug design and are primarily substrate analogs, with relatively limited related high-throughput screening and computational biology research [28]. Further exploration of the catalytic mechanisms, feedback inhibition, or allosteric inhibition mechanisms of these enzymes, combined with in vitro and in vivo infection experiments, is expected.

Among the three DapE inhibitors tested in this study, disulfiram exhibited significantly higher inhibitory activity than captopril, while penicillamine was ineffective. We hypothesize that this discrepancy may be due to differences in the hydrophilicity of the compounds. XlogP3 is a key parameter for predicting the lipophilic–hydrophilic partition coefficient (logP) of compounds: higher values indicate stronger lipophilicity and weaker hydrophilicity, while lower values indicate weaker lipophilicity and stronger hydrophilicity (PubChem: https://pubchem.ncbi.nlm.nih.gov/). *E. chaffeensis* resides in *E. chaffeensis*-containing vesicles (ECVs) in host cells and its outer membrane is rich in lipids [3,34]. Disulfiram (XlogP3 = 3.9) is likely to penetrate more efficiently than the more hydrophilic captopril (XlogP3 = 0.3) and penicillamine (XlogP3 = −1.8). Notably, the inhibitory potency of the three compounds correlated well with their lipophilicity. Thus, selecting lipophilic compounds might offer a novel strategy for the development of anti-*Ehrlichia* therapeutics. The derivatives based on disulfiram may serve as more efficacious therapeutic agents for HME in the future. Disulfiram, commercially available as Antabuse, was used to treat alcohol dependence for more than 70 years [35]. Further investigations into the optimal drug concentration, administration route, and treatment course of disulfiram for HME are warranted.

*E*. *chaffeensis* induces THP-1 cells to secrete IL-1β, IL-8, and IL-10 [7]. Here, we found that DapE specifically induces IL-8 expression. This finding implies that DapE may interact with specific receptors on THP-1 cells to trigger an inflammatory response and promote IL-8 secretion. As a key chemokine, IL-8 is primarily secreted by macrophages, and its core biological function is to recruit neutrophils, whose bactericidal activity represents an essential host innate immune response against bacterial infection [36]. In addition, several studies have reported that IL-8 is also capable of recruiting macrophages [37]. It is possible that DapE may promote the pathogenicity of *E. chaffeensis* by facilitating IL-8-mediated macrophage recruitment, thereby accelerating the progression of HME.

We found that the expression of *dapE* is regulated by NtrX. NtrX is the response regulator of the two-component system NtrY/NtrX. It has been reported that the NtrY/NtrX system senses redox changes in *Brucella abortus* [38]. In *E. chaffeensis*, the NtrY/NtrX system might detect host ROS upon bacterial infection [26]. It has been reported that NtrX regulates the expression of *putA* and *glnA*, the key enzymes of the glutamine biosynthetic pathway [25], and the expression of *argD*, the key enzyme of the arginine biosynthetic pathway [21]. These genes are closely linked in the amino acid metabolic network of *E. chaffeensis*. DapE uses L,L-SDAP as its specific substrate, whose production depends on the catalytic action of ArgD [30]. In addition, GlnA, as the core enzyme of glutamine synthesis, can synthesize glutamine using L-glutamate and ammonia, thereby maintaining the homeostasis of bacterial nitrogen metabolism [25]. Due to the reduction in the bacterial genome during evolution, *E. chaffeensis* has only a few transcriptional regulators in its genome, which results in the merging of genes into the regulons of these regulators [3]. NtrX might function as a global transcriptional regulator governing amino acid biosynthetic pathways.

## 5. Conclusions

In this study, we demonstrated that DapE is functional and essential for *E. chaffeensis* intracellular growth. Treatment with DapE inhibitors effectively reduced both *E. chaffeensis* infection and intracellular growth, with disulfiram showing substantially greater efficacy. DapE induces IL-8 expression in host cells. The expression of DapE is regulated by the two-component system NtrY/NtrX. Our findings deepen the understanding of *E. chaffeensis* pathogenesis and illustrate that DapE in *E. chaffeensis* represents a promising therapeutic target that warrants further validation.

## Data Availability

Data are contained within the article and Supplementary Materials.

## Supplementary Materials

The following supporting information can be downloaded at: https://www.mdpi.com/article/10.3390/microorganisms14091889/s1, Figure S1: The effect of penicillamine on *E. chaffeensis* intracellular growth; Figure S2: The cytotoxicity of disulfiram and captopril; Figure S3: Purification of rDapE and rNtrX DBD; Figure S4: rDapE has no significant effect on the expression of IL-1β and IL-10 in THP-1 cells; Table S1: Bacterial strains and plasmids used in this study; Table S2: Primers, PNA and ssRNA used in this study.

## Abbreviations

The following abbreviations are used in this manuscript:

HME	human monocytic ehrlichiosis
PNA	peptide nucleic acid
FBS	fetal bovine serum
LB	Luria–Bertani
CTL PNA	control PNA
cDNA	complementary DNA
qPCR	quantitative PCR
IPTG	isopropyl-thio-β-D-galactoside
SDS-PAGE	SDS-polyacrylamide gel electrophoresis
L,L-SDAP	N-succinyl-L, L-diaminopimelic acid
DAP	diaminopimelic acid
DapE	succinyl-diaminopimelic acid desuccinylase
