# Supplementary material for: Ehrlichia chaffeensis DapE Is Essential for Intracellular Growth and Represents a Promising Therapeutic Target
Source: Microorganisms. 2026 Aug 25;14(9):1889. doi: 10.3390/microorganisms14091889 (PMC13609616; doi:10.3390/microorganisms14091889)
Supplement: Supplementary file 1 [file microorganisms-14-01889-s001.zip › microorganisms-4415621-supplementary.pdf]

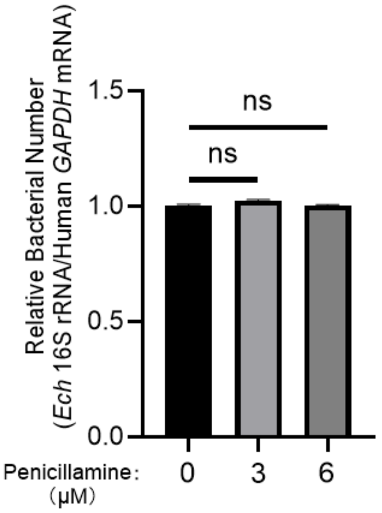

Figure S1. The effect of penicillamine on *E. chaffeensis* intracellular growth. *E. chaffeensis* infected THP-1 cells were treated with penicillamine (3 μM, 6 μM) at 37°C. At 48 h p.i., total RNA was isolated from each sample. The load of *E. chaffeensis* was quantified by qRT-PCR and normalized against human *GAPDH* mRNA. Relative levels of bacterial 16S rRNA were based on the control group. Results are presented as mean ± SD (n = 3). Statistical differences were evaluated by one-way ANOVA (ns = not significant).

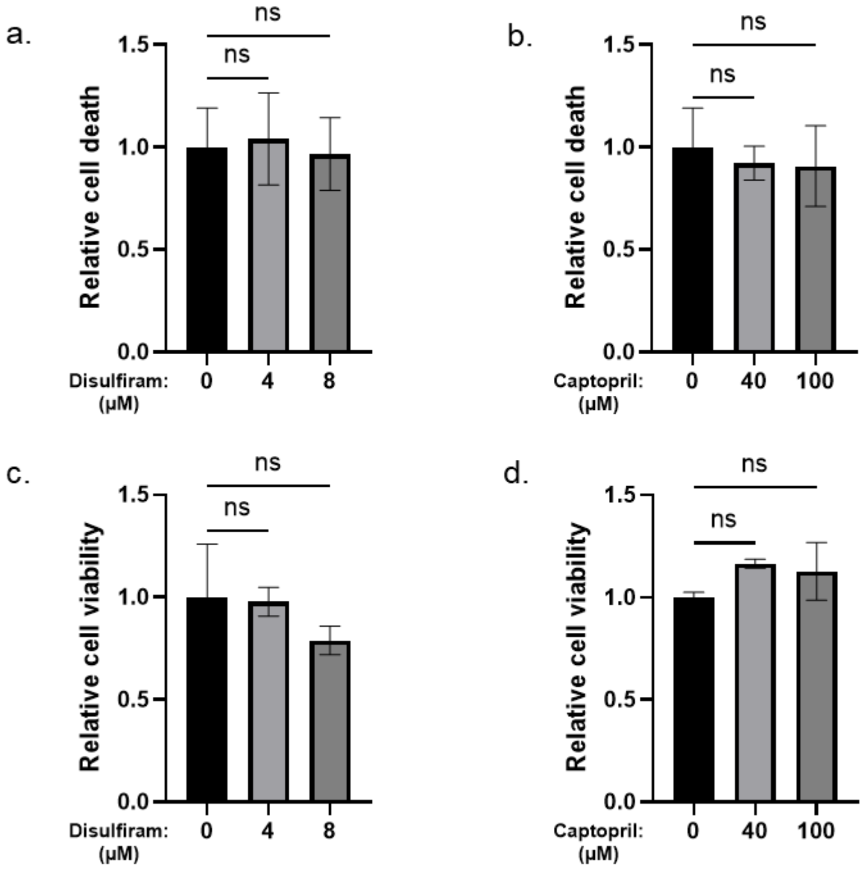

Figure S2. The cytotoxicity of disulfiram and captopril. THP-1 were seeded at a density of 4 × 10^4 cells/mL in 96-well plates and treated with the indicated concentrations of disulfiram (a, c) or captopril (b, d) at 37°C for 48 h. Cell death and viability were assessed using the Calcein-AM/PI double-staining assay. After incubation with the dyes at 37°C for 30 min in the dark, fluorescence intensity was measured. Data present relative cell death (a, b) or relative cell viability (c, d) normalized against that of the 0 μM control group. Results are presented as mean ± SD (n = 3). Statistical differences were evaluated by one-way ANOVA (ns = not significant).

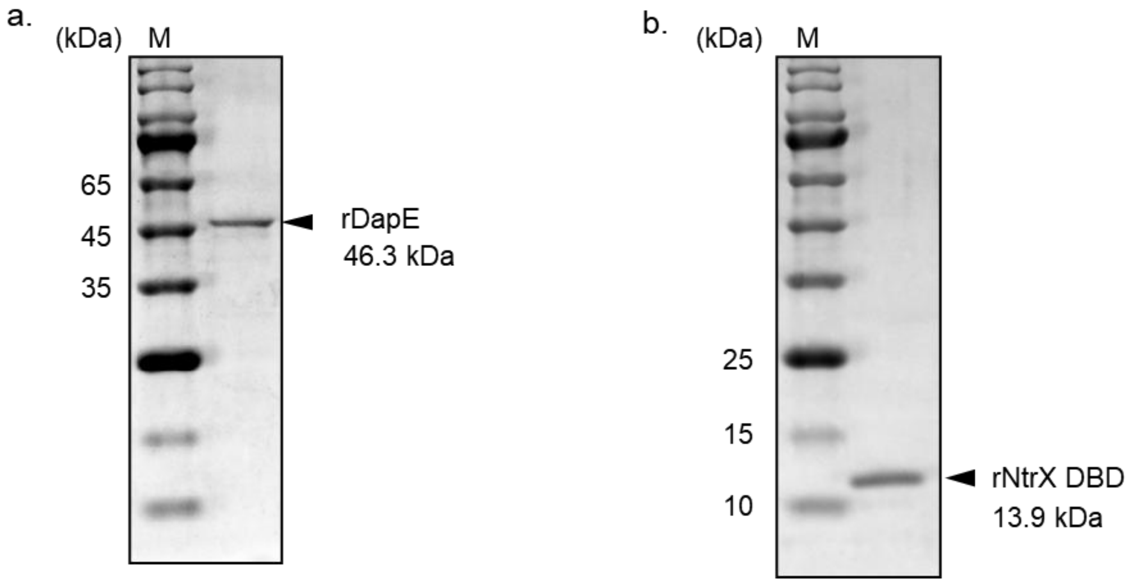

Figure S3. Purification of rDapE and rNtrX DBD. Purified rDapE (a) and rNtrX DBD (b) were separated by SDS-PAGE. The molecular masses of rDapE and rNtrX DBD are 46.3 kDa and 13.9 kDa, respectively. M: Protein molecular weight marker. Arrowheads indicate the target protein bands.

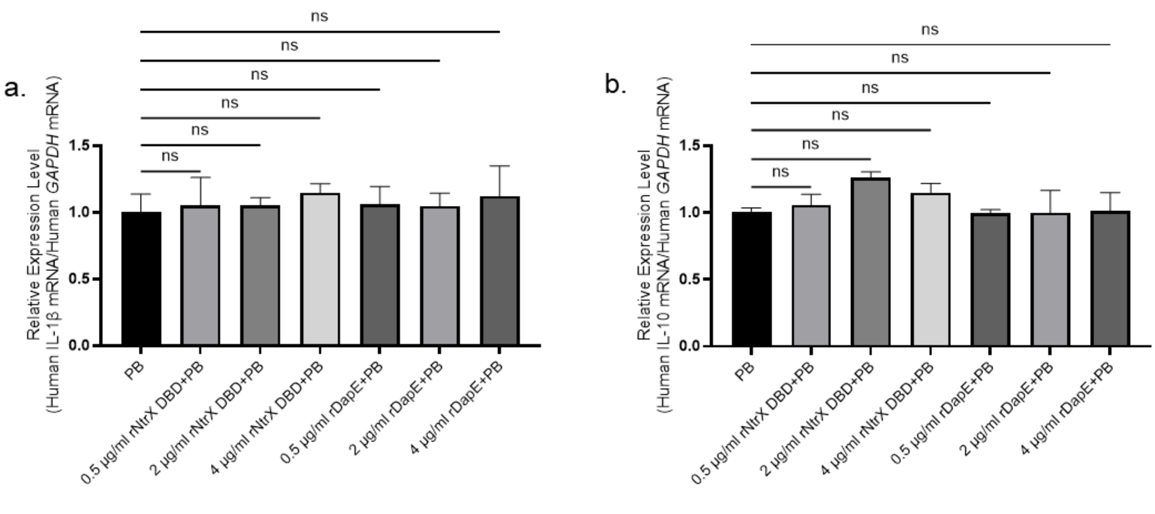

Figure S4. rDapE has no significant effect on the expression of IL-1β and IL-10 in THP-1 cells. THP-1 cells were seeded in 6-well plates at a density of 5 × 10^5 cells/mL, followed by

incubation with 20 μg/mL PB at 37°C for 24 h. Subsequently, the cells were treated with rDapE or rNtrX DBD (as a negative control) at different concentrations (0.5, 2, 4 μg/mL) at 37°C for 2 h. Total RNA was extracted. The expression levels of IL-1β (a) and IL-10 (b) were detected by qRT-PCR and normalized against those of human *GAPDH* mRNA. Relative values were based on the PB treatment group. Results are presented as mean ± SD (n = 3). Statistical differences were evaluated by one-way ANOVA (ns = not significant).

Table S1. Bacterial strains and plasmids used in this study.

Strains/Plasmids	Description	Source (Reference)
Homo sapiens THP-1		Lab stock
Ehrlichia chaffeensis Arkansas strain		Lab stock
***E. coli* strains**		
BW25113	BW25113 wild-type strain; no resistance	Lab stock
BW25113/pKD46	BW25113 harboring λ-Red recombination helper plasmid pKD46; Amp^r	Lab stock
BW25113 Δ*dapE*::Kan	BW25113 with *dapE* gene disrupted by kanamycin resistance cassette; Kan^r	This study
BW25113 Δ*dapE*::Kan/pUC19::*dapE*_Ech	BW25113 genetic complementation of *dapE*_Ech in Δ*dapE*::Kan background; Kan^r, Amp^r	Lab stock
DH5α	DH5α cloning host strain; no resistance	Lab stock
DH5α/pET-33b (+)-*dapE*	DH5α harboring pET-33b(+) plasmid carrying *dapE* gene; Kan^r	This study
DH5α/pACYC184-$dapE_{promoter}$-*lacZ*	DH5α harboring pACYC184 reporter plasmid with *dapE* promoter fused to *lacZ*; Cm^r, Tet^r	This study
BL21(DE3)	BL21(DE3) expression host strain; no resistance	Lab stock
BL21 (DE3)/pET-33b (+)-*dapE*_Ech	BL21(DE3) harboring pET-33b(+) plasmid carrying *E. chaffeensis dapE* gene for expression; Kan^r	This study
BL21 (DE3)/pET-33b (+)-*ntrX*-DBD	BL21(DE3) harboring pET-33b(+) plasmid carrying *ntrX*-DBD fusion gene; Kan^r	Lab stock
BL21 (DE3)/pET-33b (+)-*ntrX*	BL21(DE3) harboring pET-33b(+) plasmid carrying *ntrX* gene for expression; Kan^r	[25]
BL21 (DE3)/pET-33b (+)-*ntrY*-HKD	BL21(DE3) harboring pET-33b(+) plasmid carrying *ntrY*-HKD gene for expression; Kan^r	[25]
BL21 (DE3)/pET-33b (+)-*ntrX* + pACYC184-$dapE_{promoter}$-*lacZ*	BL21(DE3) co-expressing NtrX from pET-33b(+) and harboring *dapE* promoter-*lacZ* reporter; Kan^r, Cm^r, Tet^r	This study
BL21 (DE3)/pET-33b (+)-*ntrY*-HKD + pACYC184-$dapE_{promoter}$-*lacZ*	BL21(DE3) co-expressing NtrYHKD from pET-33b(+) and harboring *dapE* promoter-*lacZ* reporter; Kan^r, Cm^r, Tet^r	This study
**plasmids**		
pET-33b(+)	Expression vector; Kan^r	Lab stock
pET-33b(+)-*dapE*_Ech	pET-33b(+) harboring *E. chaffeensis dapE* gene; Kan^r	This study
pKD13	Template vector for λ-Red recombination; Kan^r	Lab stock
pUC19::*dapE*_Ech	pUC19 harboring *E. chaffeensis dapE* gene; Amp^r	This study
pACYC184-*lacZ*	Reporter vector; Cm^r, Tet^r	Lab stock

pACYC184-*dapE*$_{promoter}$-*lacZ*	pACYC184 harboring *dapE*$_{promoter}$-*lacZ* reporter; Cm^r, Tet^r	This study

Kan^r, kanamycin resistance; Amp^r, ampicillin resistance; Cm^r, chloramphenicol resistance; Tet^r, tetracycline resistance.

Table S2. Primers, PNA and ssRNA used in this study.

primers/PNA/ssRNA	sequence(5'-3')	Function
**primers**		
*dapE*_Ech-F	GGAATTCCATGGCTATTGACCCAGTAATATTAT	construct vector
*dapE*_Ech-R	CAAGCTTGTCCCTAGCACTTAGCTACTGAG	construct vector
dapE red-F	CACTCAATAGCCACTAATTCATTAAGGAAAGG TGCTGCGCGATCCGTCGACCTGCAG	PCR for Red recombination fragment
dapE red-R	GGATCAGGGCAATGAATTCTTCCCGGCGACCA GTATGCAGGAGCGATTGTGTAGGCTGGAG	PCR for Red recombination fragment
*dapE*_Ech PUC19-F	CCCAAGCTTGATGGCTATTGACCCAGTAATATT ATC	construct vector
*dapE*_Ech PUC19-R	CCCTCTAGAGTCCCTAGCACTTAGCTACTGAG	construct vector
*dapE*_Ech promoter-F	CTAAGTGCTAGGGAAAATTTTTAAC	EMSA
*dapE*_Ech promoter-R	CATAAGTTTTGACATGTAATGTATTG	EMSA
*dapE*_Ech promoter pACYC184-*lacZ*-F	CCCAAGCTTGGGCATAAGTTTTGACATGTAATG TATTG	construct vector
*dapE*_Ech promoter pACYC184-*lacZ*-R	CGCGGATCCGCGCTAAGTGCTAGGGAAAATTT TTAAC	construct vector
*dapE*_Ech qRT-F	TAATCCTAGCTCAATCACAGGAC	qRT-PCR
*dapE*_Ech qRT-R	CTGACAAATATTCTGATATGCTATC	qRT-PCR
16S rRNA(ECH)-qRT-F	GGTGAGTAATGCGTAGGAATC	qRT-PCR
16S rRNA(ECH)-qRT-R	GCTCATCTAATAGCGATAAATC	qRT-PCR
GAPDH(human)-qRT-F	TCAAGGCTGAGAACGGGAAG	qRT-PCR
GAPDH(human)-qRT-R	TGGACTCCACGACGTACTCA	qRT-PCR
IL-1β-qRT-F	GGGCCTCAAAGGAAAGAATC	qRT-PCR
IL-1β-qRT-R	TACCAGTTGGGGAACTCTGC	qRT-PCR
IL-8-qRT-F	CTGCGCCAACACAGAAATTA	qRT-PCR
IL-8-qRT-R	ATTGCATCTGGCAACCCTAC	qRT-PCR
IL-10-qRT-F	CATGGGTCTTGGGAAGAGAA	qRT-PCR
IL-10-qRT-R	AACTGGCCACAGTTTTCAGG	qRT-PCR
**PNA**		

DapE PNA	TGGCACCGTTATCTGT	DapE knockdown
**ssRNA**		
dapE ssRNA	UACUCCAACAGAUAACGGUGCCAUAGACUUU	Hybridization assay
